# Household air pollution from cooking and risk of adverse health and birth outcomes in Bangladesh: a nationwide population-based study

**DOI:** 10.1186/s12940-017-0272-y

**Published:** 2017-06-13

**Authors:** Md Nuruzzaman Khan, Cherri Zhang B. Nurs, M. Mofizul Islam, Md Rafiqul Islam, Md Mizanur Rahman

**Affiliations:** 1 Department of Population Science, Jatiya Kabi Kazi Nazrul Islam University, Mymensingh, 2220 Bangladesh; 2 Department of Global Health Policy, University of Tokyo, Tokyo, Japan; 3 Department of Public Health, La Trobe University, Melbourne, Australia; 4 Department of Population Science and Human Resource Development, University of Rajshahi, Rajshahi, 6205 Bangladesh; 5 Department of Global Health Policy, University of Tokyo, Tokyo, Japan

**Keywords:** Cooking fuel, Indoor pollution, Solid fuel, Acute respiratory infection, Low birth weight, Stillbirth, Bangladesh

## Abstract

**Background:**

Household air pollution (HAP) from cooking with solid fuels has become a leading cause of death and disability in many developing countries including Bangladesh. We assess the association between HAP and risk of selected adverse birth and maternal health outcomes.

**Methods:**

Data for this study were extracted from Bangladesh Demographic and Health Survey conducted during 2007–2014. Selected adverse birth outcomes were acute respiratory infection (ARI) among children, stillbirth, low birth weight (LBW), under-five mortality, neonatal mortality and infant mortality. Maternal pregnancy complications and cesarean delivery were considered as the adverse maternal health outcomes. Place of cooking, use of solid fuel within the house boundary and in living room were the exposure variables. To examine the association between exposure and outcome variables, we used a series of multiple logistic regression models accounted for complex survey design.

**Results:**

Around 90% of the respondents used solid fuel within the house boundary, 11% of them used solid fuel within the living room. Results of multiple regression indicated that cooking inside the house increased the risk of neonatal mortality (aOR,1.25; 95% CI, 1.02–1.52), infant mortality (aOR, 1.18; 95% CI, 1.00–1.40), ARI (aOR, 1.18; 95% CI, 1.08–1.33), LBW (aOR, 1.25; 95% CI, 1.10–1.43), and cesarean delivery (aOR,1.18; 95% CI, 1.01–1.29). Use of solid fuel, irrespective of cooking places, increased the risk of pregnancy complications (aOR, 1.36; 95% CI, 1.19–1.55). Compared to participants who reported cooking outside the house, the risk of ARI, LBW were significantly high among those who performed cooking within the house, irrespective of type of cooking fuel.

**Conclusion:**

Indoor cooking and use of solid fuel in household increase the risk of ARI, LBW, cesarean delivery, and pregnancy complication. These relationships need further investigation using more direct measures of smoke exposure and clinical measures of health outcomes. The use of clean fuels and structural improvement in household design such as provision of stove ventilation should be encouraged to reduce such adverse health consequences.

**Trail registration:**

Data related to health were collected by following the guidelines of ICF international and Bangladesh Medical Research Council. The registration number of data collection was 132,989.0.000, and the data-request was registered on March 11, 2015.

## Background

Household air pollution (HAP) arising from solid fuel use remains a global health threat. Nearly three billion of the world’s population continue to rely on solid fuel, including biomass fuels (wood, animal dung, and crop waste) to meet their energy needs [1]. Most of these people are poor and live mainly in developing countries in Asia and Sub-Saharan Africa [2]. Mainly women in these countries are engaged in cooking activities. Their newborns and kids spend a substantial amount of time with them in the kitchen. Exposure to HAP may have impact on reproductive health of women, their newborns and kids. Previous epidemiological studies found association of HAP with a range of adverse health and birth outcomes among women [3, 4]. For instance, around 34% of stroke, 26% of ischemic heart disease, 22% of chronic obstructive pulmonary disease, and 6% of lung cancer were attributed to indoor air pollution [1]. Exposure to HAP doubles the risk of pneumonia and acute lower respiratory infection, contributing to over 800,000 deaths in children under five years of age [5–7]. Around 1.6 million premature deaths and 38 million disability adjusted life-years were estimated to be associated with HAP [8]. Higher growth of population, limited access to and rising price of alternative fuel like liquefied petroleum gas influence the use of solid fuel in Asia and Africa, leading to increased burden of such adverse health outcomes [6].

In Bangladesh, where this study was conducted, majority of the population depend on several forms of solid fuel including coal, lignite, charcoal and wood [9]. Several regional studies in Bangladesh found that such practice contributes to the development of childhood pneumonia which is deemed to be one fifth of all under-five deaths [9, 10]. Exposure to HAP among women was found to be associated with several health hazards such as chronic obstetric pulmonary disease and cough [11, 12]. HAP contributes to around 4% of national burden of disease, costing 3.0% of gross national product [13, 14].

Reducing indoor air pollution and its adverse effects on preventable maternal and under-five mortality are the key targets in Sustainable Development Goal-3 [15]. Public health experts therefore became interested about HAP in general, and its associated adverse health outcomes on women and children in particular. However, studies in this area thus far were mainly limited to some specific outcomes such as acute respiratory infection, low birth weight and stillbirth [16, 17]. A few studies that were conducted in Bangladesh similarly examined only limited outcomes such as respiratory diseases among young children [18–20] and under-five mortality [21]. These studies did not consider other child and maternal health outcomes such as LBW, pregnancy complication etc., which are frequently reported in Bangladesh and other developing countries. Furthermore, most of the existing evidences that are currently available mainly focus on the consequences of solid fuel use and there is very little evidence on the impact of cooking place and indoor use of solid fuel.

Such a limited focus but continuing burden of disease calls for improvement of previous estimates on a broader scale, and the assessment of additional outcomes of maternal and child health. We, therefore, conducted an analysis of nationally representative data of Bangladesh. Demographic and Health Survey (BDHS) and examined the relationship between HAP and selected adverse maternal and perinatal outcomes. For a greater evaluation we used three different sources of HAP: (i) place of cooking, (ii) type of cooking fuel, and (iii) place of solid fuel use.

## Methods

### Data design and data source

Ever married women (aged 10–49 years) were interviewed using a stratified sample of households based on a two-stage cluster design. The detail sampling design and other related issues of BDHSs can be found elsewhere [9, 22–24]. The response rates in all waves were more than 98%. Multiple births were excluded from our analysis, as some previous studies found biological plausibility of higher mortality among this group [25, 26]. A total of 44,841 ever-married women in their reproductive age were included in three waves of dataset. Some of our selected outcome variables including neonatal, infant, and under-five mortality received limited response in the dataset of individual wave. To overcome this limitation we pooled three most recent waves of data of BDHS, collected in 2007, 2011 and 2014, to provide us the opportunity to generate a data file with sufficient statistical power. Our analysis included women (*n* = 22,789) who gave birth to live-born children within five years preceding the survey. Only outcomes of the most recent births were considered. The United States Agency for International Development provided financial support to this survey. The survey protocol was reviewed and approved by the National Research Ethics Committee in Bangladesh.

### Outcome variables

In this study we included a range of adverse reproductive health and birth outcomes. The outcome variables were child acute respiratory infection (ARI) (infection in the nose, trachea or lungs that interfere normal breathing), low birth weight (LBW) (birth weight < 2500 g), pregnancy complications (health problems during pregnancy that adversely affect the mother and/or the fetus), cesarean delivery (surgical procedure to deliver the baby), under-five mortality (the number of deaths before the fifth birthday (0–59 months)), infant mortality (the number of deaths during the first year of life (0–11 month)), neonatal mortality (the number of deaths during the first 28 days of life) and stillbirth (fetal death lasting seven or more months).

### Exposure variables

The analysis was carried out for three exposure variables: place of cooking (inside house vs outside house, irrespective of type of cooking fuel), type of cooking fuel (clean fuel vs solid fuel, irrespective of cooking place) and place of solid fuel use (indoor use of solid fuel vs no indoor use of solid fuel). During the survey the respondents were asked about the place of cooking and type of fuel they used for cooking. Clean fuel includes electricity, liquefied petroleum gas, natural gas, and biogas; and solid fuel includes coal, lignite, charcoal, wood, straw/shrubs/grass, agricultural crop, animal dung and others.

### Covariates adjustment

A range of socio-demographic variables were used in this study based on previous research demonstrating the importance of these factors [2, 20, 27, 28]. The potential covariates were women’s age at birth, wealth quintiles (poorest, poorer, middle, richest, richer), educational attainment (uneducated, primary, secondary, higher), region of residence (Barisal, Chittagong, Dhaka, Khulna, Rajshahi, Rangpur, Sylhet), place of residence (rural or urban), and children’s gender (male, female).

### Statistical analysis

We used mean and standard error to describe characteristics of the participants. We also estimated the prevalence of each outcome variable with a 95% confidence interval (95% CI). The associations between place of cooking, type of cooking fuel, and place of solid fuel use and adverse maternal and child health and birth outcomes were investigated using a series of multiple logistic regression models. The initial models included only specific outcomes and exposure variables, and the final models were adjusted for all potential confounding factors. All analyses accounted for the complex survey design. Stata software version 13.1 (Stata Corp: College Station, TX, USA) was used for all statistical analyses.

## Results

A total of 22,789 women and their children were considered eligible for this study. The total number of under-five death during the whole span of the survey is 1090, of which 740 children died within one month after birth (neonatal mortality) and 990 children died within one year after birth (infant mortality). We recorded only 564 stillbirths over the whole span of the survey period. Table 1 summarizes some basic information about the study participants, exposure and outcome variables. Solid fuel such as coal, lignite, charcoal, wood, straw/shrubs/grass, agricultural crop, and animal dung were the major (90%) cooking fuel in Bangladesh. Around 14% of the respondents performed indoor cooking, within that 11% used solid fuel.

**Table 1:** Some basic information about the study participants, exposure and outcome variables

Demographics of mothers	
Mean age (SE)	25.6 (±0.04)
Mean weight (SE)	47.8 (±0.06)
Mean year of education (SE)	5.6 (±0.03)
Exposures	
Indoor cooking place % (95% CI)	14.2 (12.7–15.8)
Use of solid cooking fuel % (95% CI)	89.8 (88.2–91.2)
Use of indoor solid fuel % (95% CI)	11.2 (9.8–12.8)
Outcomes	
Under five mortality rate per 1000 live births (95% CI)	49.0 (45.0–52.0)
Neonatal mortality rate per 1000 live births (95% CI)	34.0 (30.0–38.0)
Infant mortality rate per 1000 live births (95% CI)	45.0 (41.0–49.0)
Acute respiratory infection % (95% CI)	14.9 (14.1–15.8)
Pregnancy complication % (95% CI)	54.9 (53.0–56.7)
Low birth weight % (95% CI)	17.7 (16.6–18.8)
Cesarean delivery % (95% CI)	11.3 (10.5–12.2)
Stillbirth rate % (95% CI)	1.4 (1.2–1.5)

*SE* Standard error, *95% CI* 95% Confidence interval

Under-five mortality rate over the span of survey years was 49 per 100 live births. Neonatal and infant mortality rates were 34 and 45 per 1000 live births, respectively. In our sample a total of 2468 (17.7%) children were identified as born with LBW (<2500 g). Around 15% of children reported various symptoms of ARI. Rate of stillbirth was 1.4%. Around 55% of the women reported that they had suffered various forms of pregnancy complications during gestational period.

The results of unadjusted and adjusted models for specific health and birth outcomes are shown in Tables 2, 3 and 4. Adjusted models show that respondents performed indoor cooking reported to have significantly higher risk of neonatal (aOR, 1.25; 95% CI, 1.02–1.52) and infant mortality (aOR, 1.18; 95% CI, 1.00–1.40). None of the others two exposures showed any significant relationships with under-five mortality, neonatal mortality and infant mortality (Table 3-4). Indoor cooking and indoor use of solid fuel were found to be risk factors for developing childhood ARI after adjusting for possible confounding factors (Tables 2, 4, 5). These risks were 1.18 times (95% CI, 1.08–1.33) and 1.19 times (95% CI, 1.06–1.32) higher among the children whose mothers reported indoor cooking and used indoor solid fuel, respectively (Tables 2, 4). However, we found an insignificant association between the use of solid fuel and increased risk of ARI (Tables 3, 5).

**Table 2:** Result of the multiple logistic regression analysis assessing the association between cooking place and the risk of different adverse birth and health outcomes

Outcome variable	Cooking place		*p*
	Outside house	Inside house	
Under five mortality			
n (%)	857 (4.6)	231 (5.7)	
OR (95% CI)	1.00	1.17 (1.00–1.37)	0.05
aOR (95% CI)	1.00	1.13 (0.96–1.34)	0.14
Neonatal mortality			
n (%)	576 (3.1)	163 (4.1)	
OR (95% CI)	1.00	1.27 (1.05–1.54)	<0.05
aOR (95% CI)	1.00	1.25 (1.02–1.52)	<0.05
Infant mortality			
n (%)	774 (4.2)	215 (5.3)	
OR (95% CI)	1.00	1.22 (1.03–1.44)	<0.05
aOR (95% CI)	1.00	1.18 (1.00–1.40)	0.05
Acute respiratory infection			
n (%)	2626 (14.7)	569 (14.5)	
OR (95% CI)	1.00	1.24 (1.12–1.38)	<0.01
aOR (95% CI)	1.00	1.18 (1.08–1.33)	<0.05
Low birth weight			
n (%)	2061 (17.9)	402 (20.6)	
OR (95% CI)	1.00	1.36 (1.20–1.54)	<0.01
aOR (95% CI)	1.00	1.25 (1.10–1.43)	<0.01
Pregnancy complication			
n (%)	5168 (54.3)	938 (48.8)	
OR (95% CI)	1.00	0.77 (0.69–1.07)	0.09
aOR (95% CI)	1.00	0.78 (0.70–1.03)	0.08
Cesarean delivery			
n (%)	2410 (14.9))	498 (14.4)	
OR (95% CI)	1.00	1.24 (1.02–1.45)	<0.05
aOR (95% CI)	1.00	1.18 (1.01–1.29)	<0.05
Stillbirth			
n (%)	429 (1.17)	98 (1.22)	
OR (95% CI)	1.00	1.08 (0.85–1.37)	0.53
aOR (95% CI)	1.00	1.01 (0.90–1.28)	0.93

*n* frequency, *aOR* adjusted odds ratio (controlled for maternal age, education, place of residence, region, socio-economic status, breastfeeding and child sex)

**Table 3:** Result of the multiple logistic regression analysis assessing the association between types of cooking fuel and the risk of different adverse birth and health outcomes

Outcome variable	Types of cooking fuel		*p*
	Clean fuel	Solid fuel	
Under five mortality			
n (%)	213 (5.5)	875 (4.6)	
OR (95% CI)	1.00	0.92 (0.76–1.10)	0.32
aOR (95% CI)	1.00	0.96 (0.78–1.19)	0.71
Neonatal mortality			
n (%)	139 (3.6)	600 (3.2)	
OR (95% CI)	1.00	0.94 (0.75–1.17)	0.58
aOR (95% CI)	1.00	1.03 (0.80–1.33)	0.80
Infant mortality			
n (%)	198 (5.1)	791 (4.2)	
OR (95% CI)	1.00	0.98 (0.79–1.22)	0.87
aOR (95% CI)	1.00	0.88 (0.73–1.07)	0.20
Acute respiratory infection			
n (%)	530 (14.4)	2667 (14.8)	
OR (95% CI)	1.00	1.16 (1.02–1.33)	<0.05
aOR (95% CI)	1.00	1.07 (0.95–1.20)	0.29
Low birth weight			
n (%)	328 (20.9)	2140 (18.0)	
OR (95% CI)	1.00	0.88 (0.76–1.02))	0.10
aOR (95% CI)	1.00	0.96 (0.81–1.13)	0.63
Pregnancy complication			
n (%)	818 (45.7)	5289 (54.7)	
OR (95% CI)	1.00	1.33 (1.19–1.49)	<0.01
aOR (95% CI)	1.00	1.36 (1.19–1.55)	<0.01
Cesarean delivery			
n (%)	431 (13.8)	2479 (15.1)	
OR (95% CI)	1.00	1.29 (1.01–1.64)	<0.05
aOR (95% CI)	1.00	1.24 (0.95–1.60)	0.11
Stillbirth			
n (%)	69 (1.1))	458 (1.2)	
OR (95% CI)	1.00	1.33 (1.02–1.82)	<0.05
aOR (95% CI)	1.00	1.09 (0.86–1.37)	0.53

*n* frequency, *aOR* adjusted odds ratio (controlled for maternal age, education, place of residence, region, socio-economic status, breastfeeding and child sex)

**Table 4:** Result of the multiple logistic regression analysis assessing the association between types of indoor cooking fuel and the risk of different adverse birth and health outcomes

Outcome variable	Place of use solid fuel		*p*
	No indoor solid fuel	Use indoor solid fuel	
Under five mortality			
n (%)	741 (4.5)	134 (5.5)	
OR (95% CI)	1.00	1.17 (0.97–1.43)	0.11
aOR (95% CI)	1.00	1.11 (0.91–1.35)	0.29
Neonatal mortality			
n (%)	503 (3.1)	97 (4.1)	
OR (95% CI)	1.00	1.28 (1.02–1.61)	<0.05
aOR (95% CI)	1.00	1.23 (0.97–1.55)	0.08
Infant mortality			
n (%)	667 (4.1)	124 (5.2)	
OR (95% CI)	1.00	1.23 (1.00–1.50)	0.05
aOR (95% CI)	1.00	1.15 (0.94–1.42)	0.17
Acute respiratory infection			
n (%)	2318 (14.8)	347 (15.2)	
OR (95% CI)	1.00	1.26 (1.11–1.45)	<0.05
aOR (95% CI)	1.00	1.19 (1.06–1.32)	0.01
Low birth weight			
n (%)	1865 (17.6)	270 (21.2)	
OR (95% CI)	1.00	1.47 (1.26–1.70)	<0.01
aOR (95% CI)	1.00	1.33 (1.14–1.56)	<0.01
Pregnancy complication			
n (%)	4718 (55.4)	569 (49.8)	
OR (95% CI)	1.00	0.72 (0.64–1.06)	0.10
aOR (95% CI)	1.00	0.71 (0.63–1.03)	0.07
Cesarean delivery			
n (%)	2196 (15.3)	281 (13.1)	
OR (95% CI)	1.00	0.80 (0.61–1.02)	0.06
aOR (95% CI)	1.00	0.88 (0.76–1.04)	0.12
Stillbirth			
n (%)	391 (1.7)	67 (1.3)	
OR (95% CI)	1.00	1.12 (0.86–1.47)	0.39
aOR (95% CI)	1.00	0.96 (0.73–1.27)	0.78

*n* frequency, *aOR* adjusted odds ratio (controlled for maternal age, education, place of residence, region, socio-economic status, breastfeeding and child sex)

**Table 5:** The relationship between exposure and selective outcome variables in terms of significance level

Exposure variable	ARI	LBW	Pregnancy complication	Cesarean delivery
Indoor cooking (irrespective of fuel type) - Ref: outdoor cooking	<0.05	<0.01	Insignificant	<0.05
Use of solid fuel (irrespective of location of cooking) - Ref: clean fuel	Insignificant	Insignificant	<0.01	Insignificant
Indoor use of solid fuel - Ref: no use of indoor solid fuel	<0.05	<0.01	Insignificant	Insignificant

Controlled for maternal age, education, place of residence, region, socio-economic status, and breastfeeding and child sex. Outcome variables that were found significant with at least one of the exposure variables were reflected in this table

Respondents who performed indoor cooking reported to have significantly higher risk (aOR, 1.25; 95% CI, 1.10–1.43) of LBW than those who reported outdoor cooking. This risk was significantly higher (aOR, 1.33; 95% CI,1.14–1.56) among women who reported indoor use of solid fuel than others. Respondents who used solid fuel (irrespective of location of cooking) were more likely to report pregnancy complications (aOR, 1.36; 95% CI, 1.19–1.55) than those who did not use solid fuel. There was an association between cooking place and cesarean delivery (aOR, 1.18; 95% CI, 1.01–1.29). This risk was elevated (marginally insignificant) for mothers who used solid fuel compared to those who used clean fuel (aOR, 1.24; 95% CI, 0.95–1.60). None of the three exposure variables were significantly associated with stillbirth (Table 5).

## Discussion

Using national data in Bangladesh this study assessed the effect of household air pollution from cooking activities on selected adverse birth and maternal health outcomes. Our findings suggest that majority of the respondents used solid fuel. We found that both *cooking inside the house* and *indoor use of solid fuel* were significant risk factors of cesarean delivery, ARI and LBW. Indoor cooking place also increased the risk of neonatal and infant mortality. Additionally, we found solid fuel use was a significant risk factor for pregnancy complication, stillbirth and elevated risk of cesarean delivery.

**Fig. 1:**
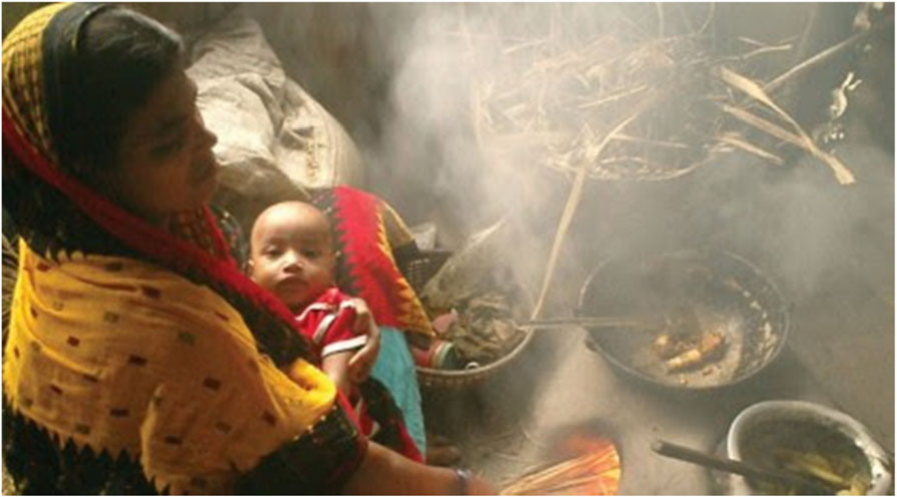
A typical kitchen in rural households of South Asia. The photograph was taken in August 2004 in Bangladesh. Photographer: Prabir Mallik. Reproduced by permission of World Bank (https://creativecommons.org/licenses/by/3.0/igo)

Globally ARI is a leading cause of death in children and its association with HAP has been well established [16, 28]. Around 3.5% of the worldwide burden of disease for under-five children and 15% of total under-five mortality are associated with ARI [29]. This number reaches to 40% if neonatal pneumonia is included [30]. Around 70% of such adverse outcomes occur in 15 Southeast Asian and African countries where HAP from cooking activities are common [31, 32]. Previous studies in these countries reported solid fuel use is a major risk factor for ARI, and the findings are similar to ours [10, 21, 30]. However, none of those studies considered the role of cooking place on developing childhood ARI. Our study demonstrates the strong effects of cooking place and indoor use of solid fuel on developing childhood ARI. Children under the age of five, especially neonates and infants, are exposed to such pollutants because they often spend time with their mothers while they are engaged in cooking activities (Fig. 1). In addition, small apartment/house oriented life structure and indoor based life of children under-five may contribute to higher levels of exposure to air pollution, leading to the development of ARI. This finding is biologically plausible as polluted cooking fuel contains a range of key pollutants including carbon mono-oxide, oxide of nitrogen and sulfur, benzene formaldehyde, 1,3-butadiene, and polyaromatic compound [21].

We also found *indoor cooking place* and *indoor use of solid fuel* increased the risk of LBW. Our finding are consistent with the results of a meta-analysis and of a study conducted based on NHFS-3 data [17, 33, 34]. However, those studies considered a broad spectrum of air pollution including cigarette smoking, use of motor vehicles along with HAP from cooking activities. An incomplete combustion, which is likely to occur in indoor cooking using solid fuel, may result in much of the fuel energy being emitted as potentially toxic pollutants including higher levels of carbon-monoxide. The fetus is particularly vulnerable to the transmission of such pollutants that a pregnant woman inhales from the living spaces. These kind of pollutants also reduce the oxygen carrying capacity of blood to the body tissue [33]. Thus a developing fetus can be deprived of adequate oxygen, leading to intrauterine growth retardation and risk of LBW [35].

In the ‘global burden of disease study’ HAP was ranked the second major contributor to the burden of diseases, after unsafe water and sanitation [36]. Around 3% of the global burden of disease and 5% of loss of healthy life years were attributed to HAP-associated illnesses [37]. A recent meta-analysis found that HAP from cooking was a major risk factor for developing tuberculosis, which is responsible for 1.4 million deaths worldwide [38, 39]. Some epidemiological studies also provided evidence that solid fuel is a risk factor for obstructive pulmonary disease, lung cancer, and heart disease [40–43]. However, none of the studies evaluated the health impact of HAP exposure during gestational time. In this study, we have been able to demonstrate the effects of HAP exposure on pregnancy complications, particularly for women performing cooking activities using solid fuel. Recent studies in the United States [3, 4] and Sweden [44] found higher risk of gestational diabetes, gestational hypertension, lower gestation age and preeclampsia among solid fuel users. Regular and/or lengthier exposure to several emitting pollutants from solid fuel during cooking could be risk factors of such complication.

Our results suggest that out of the three exposure variables, effects of *indoor cooking* (irrespective of the fuel type) and *indoor use of solid fuel* are similar. One possible explanation is that the subset of samples in these two groups are largely similar in that both of these groups reported indoor cooking; over 78% of the respondents who reported *indoor cooking* also reported *indoor use of solid fuel*. Interestingly, effects of the other exposure variable *– use of solid fuel* (irrespective of the location of cooking) – is quite different (Table 5). This is possibly due to the fact that the effect of fumes from solid fuel are substantially more harmful when they are inhaled during indoor cooking. The possible reason for an insignificant association between *use of solid fuel* (irrespective of the location of cooking) and ARI, LBW or cesarean delivery is that the vast majority of those who reported *use of solid fuel* reported outdoor cooking (90%). This over representation of outdoor cooking diluted the effect of such association. When we compare groups who *used solid fuel* vs *clean fuel* we possibly compare two different groups – relatively poor vs the well-off. Although we have adjusted this association for possible covariates such as socio-economic status; however, this might not be enough to address this difference.

Our study has a number of strengths. Firstly, we used a relatively large sample from a nationally representative study population. Secondly, the response rate was satisfactorily high (98%). Thirdly, unlike previous studies we considered a range of adverse health outcomes. Fourthly, we analyzed the data in multivariate framework by considering the survey design, which produced reliable results. However, the study’s cross-sectional nature meant that it was not possible to establish a causal relationship between the exposure and outcome variable. Also data were self-reported and it was not possible by interviewers to validate the responses. Since 2011 the Demographic and Health Survey in developing countries retrospectively collected mother’s recall of size at birth as the proxy to birth weight, which may cause some misclassifications. However, several studies [45–47] were conducted using this data and a study in Bangladesh found that the estimate is accurate for 90% of those newborns who had LBW [46]. As we used pooled data the average rates of some variables fluctuated from the rates for individual years. However, this may be considered both as a limitation and a strength. Finally, the number of observations fluctuated for some outcome variables, as they were introduced only in the recent two surveys. However, our results are not influenced by this, as we used separate model for each outcome.

## Conclusion

Indoor cooking, use of solid fuel for cooking, and indoor use of solid fuel are risk factors for a wide range of adverse pregnancy and birth outcomes. These results are important and suggest performing cooking activities outside the household without using solid fuel. Shifting cooking place to outdoor setting, having necessary stove ventilation and replacing indoor use of solid fuel should be recommended and encouraged. These recommended practices are likely to reduce child mortality and morbidity, adverse pregnancy and birth outcomes. Findings of this study could help to design health policies and health initiatives to improve maternal and child health.

## Abbreviations

ARI: Acute respiratory infection
BDHS: Bangladesh demographic and health survey
CI: Confidence interval
HAP: Household air pollution
LBW: Low birth weight
